# *Trypanosoma cruzi* Clone Dm28c Draft Genome Sequence

**DOI:** 10.1128/genomeA.01114-13

**Published:** 2014-01-30

**Authors:** Edmundo Carlos Grisard, Santuza Maria Ribeiro Teixeira, Luiz Gonzaga Paula de Almeida, Patricia Hermes Stoco, Alexandra Lehmkuhl Gerber, Carlos Talavera-López, Oberdan Cunha Lima, Björn Andersson, Ana Tereza R. de Vasconcelos

**Affiliations:** a Departamento de Microbiologia, Imunologia e Parasitologia, Universidade Federal de Santa Catarina, Florianópolis, Brazil; b Departamento de Bioquímica e Imunologia, Universidade Federal de Minas Gerais, Belo Horizonte, Brazil; c Laboratório de Bioinformática, Laboratório Nacional de Computação Científica, Petrópolis, Brazil; d Department of Cell and Molecular Biology, Karolinska Institutet, Stockholm, Sweden

## Abstract

*Trypanosoma cruzi* affects millions of people worldwide. Clinical variability of Chagas disease can be due to the genetic variability of this parasite, requiring further genome studies. Here we report the genome sequence of the *T. cruzi* Dm28c clone (TcI), a strain related to the sylvatic cycle of the parasite.

## GENOME ANNOUNCEMENT

*Trypanosoma cruzi* is a protozoan parasite that causes Chagas disease, an illness that affects 8 to 10 million people in Latin America. The genetic variability found in the parasite population allows classification of strains into six major groups (TcI through TcVI) (1). In 2005, the *T. cruzi* CL Brener clone genome was published (2), but its complete assembly was hindered by repeated sequences and the hybrid nature of this strain. Other genomes, such as the Sylvio X10/1 strain, have been published (3), allowing comparative analyses to assess the variable clinical forms of Chagas disease. Whereas CL Brener (TcVI), together with the TcII and TcV strains, is the predominant cause of Chagas disease in the Southern Cone of Latin America (1, 2), Sylvio X10/1 and Dm28c belong to TcI, a group related to the sylvatic cycle that is also associated with Chagas disease in the Brazilian Amazon, Venezuela, Colombia, and Central America (3). Here, we present the genome sequence of the *T. cruzi* clone Dm28c (4), which has been widely used as a model for biochemistry, cell biology, immunology, gene expression, and drug development studies.

Sequencing was carried out using the 454 GS-FLX Titanium platform. Two shotgun libraries and one 3-kb paired-end (PE) library were constructed using ±5 µg of Dm28c DNA isolated from epimastigote cultures. Library construction, titration, emulsion PCR, and sequencing steps were performed according to the manufacturer’s protocol. Assembly of the Dm28c genome was carried out by processing the 454 SFF files using custom Perl scripts to generate paired-end (PE) FASTQ files. The SFF files were subsequently assembled using the Newbler software and the output was scaffolded using SSPACE 2.1.0. Gaps were improved by use of GapFiller 1.11 prior to automated annotation. Using this protocol, a total number of 5,287 contigs were generated, and 91.7% of them (4,848) were assembled into 1,378 scaffolds, totaling 30,716,540 bp. The longest contig was 89,747 bp.

Automated functional annotation was performed *de novo* using the System for Automated Bacterial Integrated Annotation (SABIA) (5). We identified 4,144 proteins with homology to known proteins from other organisms as well as 7,254 hypothetical proteins, with a coding sequence (CDS) average length of 1,397 bp. With an estimated haploid genome size of 27 Mbp and a G+C content of 53.26%, Dm28c is more similar, as expected, to Sylvio X10/1 (98.71% nucleotide sequence identity) than to CL Brener (90.20%), as revealed by a stringent Megablast analysis of both coding and noncoding regions. Accordingly, the Bidirectional Best Hit (BBH) analysis showed that DM28c has 6,094 proteins with best hits with SylvioX-10 and 5,267 proteins with best hits with CL Brener. The assembled genome together with the functional annotation is available on http://www.labinfo.lncc.br/index.php/trypanosoma-cruzi-dm28.

### Nucleotide sequence accession number.

The *T. cruzi* Dm28c genome sequence is available in GenBank with the accession number AYLP00000000.
